# A 2D model to study how secondary growth affects the self-supporting behaviour of climbing plants

**DOI:** 10.1371/journal.pcbi.1011538

**Published:** 2023-10-16

**Authors:** Giacomo Vecchiato, Tom Hattermann, Michele Palladino, Fabio Tedone, Patrick Heuret, Nick P. Rowe, Pierangelo Marcati

**Affiliations:** 1 Gran Sasso Science Institute, L’Aquila, Italy; 2 AMAP, Univ Montpellier, CIRAD, CNRS, INRAe, IRD, Montpellier, France; 3 Department of Information Engineering, Computer Science and Mathematics, University of L’Aquila, L’Aquila, Italy; University of California, Riverside, UNITED STATES

## Abstract

Climbing plants exhibit specialized shoots, called “searchers”, to cross spaces and alternate between spatially discontinuous supports in their natural habitats. To achieve this task, searcher shoots combine both primary and secondary growth processes of their stems in order to support, orientate and explore their extensional growth into the environment. Currently, there is an increasing interest in developing models to describe plant growth and posture. However, the interactions between the sensing activity (e.g. photo-, gravi-, proprioceptive sensing) and the elastic responses are not yet fully understood. Here, we aim to model the extension and rigidification of searcher shoots. Our model defines variations in the radius (and consequently in mass distribution) along the shoot based on experimental data collected in natural habitats of two climbing species: *Trachelospermum jasminoides* (Lindl.) Lem. and *Condylocarpon guianense* Desf.. Using this framework, we predicted the sensory aspect of a plant, that is, the plant’s response to external stimuli, and the plant’s proprioception, that is, the plant’s “self-awareness”. The results suggest that the inclusion of the secondary growth in a model is fundamental to predict the postural development and self-supporting growth phase of shoots in climbing plants.

## Author summary

Plant growth is influenced by many external and internal factors, such as light, gravity, structural flexibility of the stem or hormone fluxes. In recent years, plant movements and modelization have received an increasing attention, leading to a better understanding of plant development. In this work, we introduce a 2D model for self-supporting structures developed by climbing plants. This model is in the direction of filling the gap that currently exists between models for plant sensing activity and models focused on the mechanical aspect of plant growth. Indeed, we consider the response of the plant to external cues together with the capability of the plant to perceive itself (proprioception) and the radial expansion process (secondary growth). We then see how to retrieve the model parameters from a minimal set of experimental data and finally test the model by comparing its numerical simulations with real plant shapes. Our result shows that a better consideration of mass distribution along the shoot is important to understand the shape of self-supporting structures.

## Introduction

The seminal work of Charles Darwin (1865) on the support-searching movements of climbing plants opened up new paths of thinking on plant life histories. Known to be the longest plants on land, climbing plants exhibit a wide diversity of mechanical architectures and ecological strategies [1] across many different phylogenetic groups [2]. Unlike trees that remain self-supporting throughout their entire life cycle, climbing plants are well-known for relying on physical support to grow vertically and towards light. To exploit supports, climbing plants rely on a developmental phase, known as the “searcher shoot”, which is specialized in crossing gaps, and searching and attaching to supports [3, 4]. To achieve these tasks, searcher shoots combine primary and secondary growth processes and actively modulate their development according to internal and external stimuli [5]. For example, it is well known how the contact with a support induces specific growth responses (i.e. thigmomorphogenesis), but it remains poorly understood how the searcher shoot respond and interact with gravity during different kinds of exploratory tasks, such as growing in a specific direction or exploring volumes by circumnutational movements.

### Plant shape and movement as a stimuli-response phenomenon

The responses that led to a turning movement and alignment of growth of a plant organ with respect to external vectorial cues have been known for many years as *tropisms* [6, 7]. A plant organ may tend to orient or grow towards a stimulus (positive tropism), or away from it (negative tropism) [8]. For example, many aerial shoots of plants grow upwards against gravity whereas most roots tend to grow downwards [7] to locate the ground. Both organs perceive gravity via modified plastids (statholits) within specialised cells (statocytes), but the growth responses are induced by different biochemical signalling and auxin flux carriers [9, 10]. Supported by mathematical analysis and models, the gravitational responses have been linked with the statholits sedimentation, as well as the diffusion of auxin, and they are considered in terms of differential growth within the plant body [11–14]. Given the complexity of interaction between the growing activity of plants and external and internal cues, growth movements can be viewed as an integrative response to diverse specific stimuli-response processes.

### Brief review of mathematical models

One of the earliest studies investigating the stimuli-response behaviour in plants was carried out by Julius Sachs in 1882 [15]. As a result of his observations, the formation of curvatures along the plant body against gravity had first only been linked to gravity perception during primary growth. The upward bending movement of the shoot being stronger in the horizontal position then the vertical one has been described according to the “sine law” because it can be reformulated with the relation ∂_*t*_*κ* ∝ sin *θ*. Here ∂_*t*_*κ* is the curvature change rate and *θ* is the inclination of the stem. This fundamental relation between curvature and inclination has remained in the biological cultural framework for over a century. Only in 2013 the mathematical studies of Bastien et al. [16] demonstrated that if the phenomenon of the response to gravity is described only on the base of the sine law, the plant can never reach a vertical steady state. This is due to infinite lateral oscillations that would arise during the upward growth of the shoot. To stabilise the self-supporting system, the sine law was modified with a positive proprioceptive term, becoming ∂_*t*_*κ* ≈ sin *θ* − *κ*. This additional term tends to regulate high curvature *κ* towards 0 in time.

In parallel with the sensing activity of the plants, a wide literature has been developed on the physical and mechanical properties of shoot growth [17–23]. Knowledge of developmental changes and physical parameters, such as diameter, length and stiffness have been considered as the main descriptors of the stem shape. Founded on the Euler-Bernoulli beam theory, these mathematical models assume that a plant shoot behaves as a growing elastic rod, and they are mainly based on two configurations: (i) the current configuration, which corresponds to the actual shape of the elastic rod when subject to gravity or any external forces, and (ii) the relaxed or intrinsic configuration, that is the shape of the rod in the weightless limit case. In particular, in case (ii), the shape of the rod is described by a purely geometric evolution equation, which neglects mechanical effects. Further details about these configurations are given in section.

Recent studies have also combined analysis on gravitropism and proprioception along with analyses on the mechanics of growth, including a planar model of a growing plant [24]. Here the elastic rod is subject to the effects of gravity and develops according to the sine law corrected via a proprioceptive term as proposed in [16]. Some excellent guidelines in the modelisation of the sensing activity and its interaction with the plant growth mechanics can be found in [25–27].

In these models the formation and the growth of tissue layers, resulting from the secondary growth, together with the development of the intrinsic curvature in function of the shoot inclination and the proprioception have never been considered [27]. For example, some models have included a linear density parameter *ρ*, which is constant along the stem and doesn’t change over time (for instance, [11, 24]). This potentially limits two important features characterising actual shoot growth: first, it assumes that the shape, the size of the stem cross-section and any internal growth expansion of mechanical tissue (e.g., of the wood cylinder) remain constant over time. Second, it does not take into account the build-up of mass along the shoot due to secondary growth of the wood cylinder and other tissues. Early development of additive growth, maturation and stiffening of mechanical tissue via early secondary growth are key development features in plant shoots in general and especially in young searcher shoots of climbing plants [28–30]. Stem stiffness and rigidity can be significantly modified by even small changes of expansion of the wood cylinder within the primary body of the plant stem even before noticeable changes in external stem diameter [3]. However, this is only one of the possible ways in which a searcher shoot can adjust its mechanical properties. From its base to the apex, a shoot can adjust its rigidity by decreasing the radius, modifying the structure and the chemistry of tissues [31] or modifying the gradient of tissue stiffness along the stem. The complexity of the interaction between all these mechanisms likely leads to a very complicated gradient of organisation, which is arguably difficult to capture or integrate using a single unifying model.

### Purpose of the study

The aim of this study is to display the relevance of the mechanics in the behaviour of a climbing plant searcher shoot, considering in particular the radial expansion of the main stem. To this end, we first develop a mathematical model able to capture a variety of shapes and orientations observed in climbing plant searchers; second, we develop an approach to reconstruct extensional growth against gravity from a static description of the shoot final state. This approach aims to use a minimal number of parameters, which can be relatively easily obtained from selected field observations of different species with variable behaviours. In particular, we aim to show how the interplay between variable linear density, proprioception and external stimuli can generate variable shapes and orientations of searcher shoots observed in two different climbing plants species in the family of Apocynaceae: *Trachelospermum jasminoides* (Lindl.) Lem. and *Condylocarpon guianense* Desf. These relatively close-related species have been chosen because they share some fundamental properties during their searching and twining growth behaviour. Both species (i) attach to support by twining; (ii) are capable of reaching similar maximal reach capacities of around 110 *cm* in length (iii) and have similar values of structural Young’s modulus at the base of searcher shoots of around 3000 *N m*^−2^ [29, 30].

In order to model a generically directed stimulus we consider the equations used by Guillon et al. [21] corrected with the proprioceptive term introduced by Bastien et al. [16]. The resulting growth dynamics is addressed through numerical simulations. Some recent studies have used numerical tools based on arbitrary parameters to illustrate generic mechanical behaviours through simulations [11, 12, 24]. Rather than arbitrarily calibrating every parameter, we set their order of magnitude using measured data from the two climbing plant species *T. jasminoides* and *C. guianense* growing in natural conditions. This enabled us to calibrate the morphological parameters as well as the measurements of internode length at different times, which were crucial for estimating the growth parameters.

### Glossary

In this study, we use some technical terms that might not be familiar or that need clarification for a diverse scientific community. To avoid confusion, we present a short glossary here.

**Searcher shoot**: Part of a climbing plant responsible for spanning gaps, foraging for support and attaching. A searcher shoot always includes a main axial structure (most often a stem) which is mechanically self-supporting from the base. According to the species, it may bear different structures such as leaves, and branches, as well as structures modified into attachment systems such as tendrils and stem segments capable of twining. Searcher stems can often undergo growth-induced movements specialized in exploring its vicinity and support attachment.

**Fig 1:**
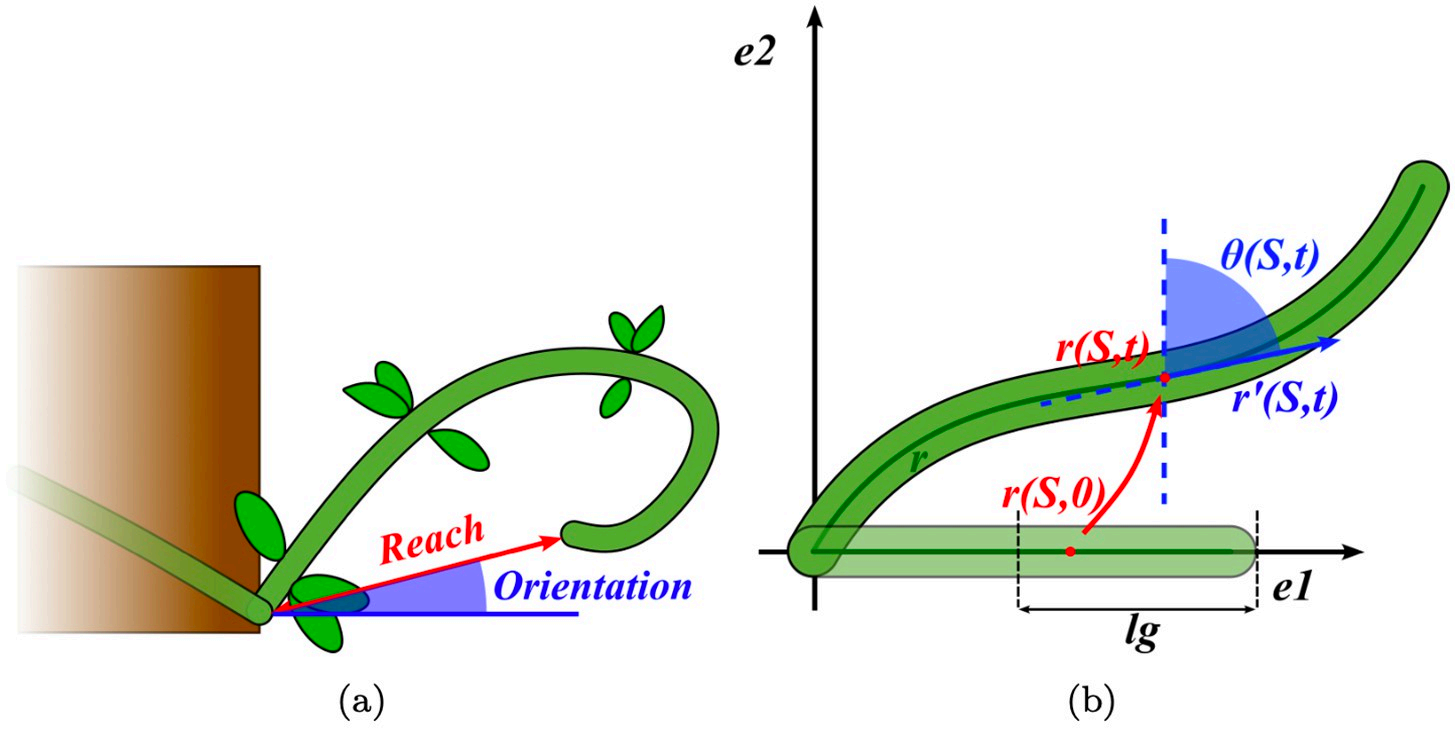
(1A): **Reach and orientation in searcher stems**. Measurement of reach and orientation in a typical searcher shoot of a climbing plant. The reach is measured as a straight line form the base of the searcher stem at its point of attachment with the parent bearing stem to the curved hook-like apex. It represents the effective distance a self-supporting searcher stem often capable of movement towards the apex. The orientation is the slope of that joining line with respect to the horizontal line. (1B): **Schematic illustration of the curve elaborated by the mathematical model**. At each time *t* of searcher shoot growth, the mathematical model represents its position in the space with a curve *r*. In the figure, ***e***_**1**_ and ***e***_**2**_ are the two orthogonal vectors that span the plane in which the curve is confined. So, these vectors correspond respectively to the (*x*, *y*) coordinates of the curve. For a given time *t*, any point of the curve is identified with the vector ***r***(*S, t*), where *S* is a parameter which varies in [0, *ℓ*_0_]. The figure shows that the mathematical model leads the point of the curve at time zero with position ***r***(*S*, 0) to the point at time *t* with position ***r***(*S, t*). Each point ***r***(*S, t*) of the curve has a certain inclination to the vertical line, denoted by *θ*(*S, t*). *ℓ*_*g*_ is the parameter used by the model as length of the extension zone. The sensing equation and the growth involve only the points of that zone.

**Reach**: The effective length observed between the apical tip of a searcher shoot and the basal point from which it is attached or fixed (see Fig 1). Maximal reach can be viewed as a functional descriptor of searcher-shoot gap-spanning capacity in a functional and ecological context.

**Orientation**: The slope of the line joining the base with the tip of a searcher stem in a vertical plane. Here, the orientation was measured only at the final configuration when the searcher shoot was estimated to have attained its maximal reach in a self-supporting state (see Fig 1).

**Primary Growth**: The increase in growth results from cell division in the apical meristem of axes and subsequent cell elongation and maturation. These lead to the growth in length of the searcher stem. In this paper we use the term *extension* as the morphogenetic additive process leading to the “lengthening” of a shoot during primary growth. Hence, the term describes a macroscopic phenomenon that implicitly includes smaller-scale growth processes such as: cell initiation, multiplication, differentiation and maturation.

**Secondary Growth**: The increase in growth that results from cell division in the vascular cambium; a ring-like meristematic tissue producing wood (secondary xylem and ray tissue) and secondary phloem. Subsequent expansion and maturation of additionally formed cells lead to a radial thickening of the stem. We use the term *rigidification* to refer to the process of stem thickening and stiffening that is achieved by the stem during secondary growth.

**Proprioception**: The capability of the shoot to perceive changes in shape and orientation in terms of curvature and to respond to these changes in order to restore local straightness.

## Materials and methods

### Formulation of the model: Mechanics and growth

#### Notations for modelling a planar morphoelastic rod

We model the searcher shoot using the theory of morphoelastic rods [32]. Hence, we are assuming that climbing plants’ searcher shoot behaves like an elastic rod that at each time *t* changes its length and shape. In this framework, we make the following assumptions: (i) at each time *t* ∈ [0, *T*], where *t* = 0 and *t* = *T* are respectively the initial and the final time of the shoot’s development, the elastic rod’s centreline is confined in a plane spanned by two orthonormal vectors {**e**_1_, **e**_2_}; (ii) the rod is inextensible and unshearable. With these premises, the rod’s model is characterised by three elements (which have been partially introduced in Section:

1. **The intrinsic configuration at the initial time** *t* = 0 (also called *reference* configuration). We assume that this configuration is just a straight line along **e**_1_ with arc-length parameter *S* ∈ [0, *ℓ*_0_];
2. **The current configuration at a generic time *t***. We denote the corresponding centreline curve with **r**(*s*, *t*) ∈ span{**e**_1_, **e**_2_}, where *s* ∈ [0, *ℓ*(*t*)] is the arc-length parameter. It is convenient to describe the position of this curve using the angle *θ*(*s*, *t*) between the tangent vector ∂_*s*_**r**(*s*, *t*) and the vector **e**_2_ (see Fig 1). The symbol ∂_*s*_ denotes the partial derivative with respect to the parameter *s* (a similar notation is used for ∂_*t*_ and ∂_*S*_). Hence, the quantity ∂_*s*_*θ*(*s*, *t*) gives the curvature of this configuration;
3. **The intrinsic configuration at a generic time *t***. We only need to fix two notations about this configuration: (i) Since the rod is inextensible, this configuration has the same arc-length parameter *s* ∈ [0, *ℓ*(*t*)] of the current configuration; (ii) we indicate with *κ*_*i*_(*s*, *t*) the curvature.

#### Equations of balance with the gravity force

We assume that the only force acting on the rod is the gravity. Using the *Kichhoff* equations for the elastic rod in the static case (see [32] or Section A in S1 Text for further details), we get

$$\begin{array}{c} \begin{array}{cc} {}[\int_{s}^{\ell (t)}\rho \left(\sigma ,t\right)d\sigma +m_{l}\left(s,t\right)]g\;\text{sin}\;\theta \left(s,t\right) & =\partial_{s}\left[B\left(s,t\right)\left(\kappa \left(s,t\right)-\partial_{s}\theta \left(s,t\right)\right)\right] \end{array}. \end{array}$$ (1)

This relation expresses the balance of gravity with the internal forces and moments (of force) developed by the rod in response. In the notation of Eq (1), *g* is the gravity acceleration constant, *B*(*s*, *t*) the flexural rigidity of the rod at **r**(*s*, *t*), *m*_*l*_(*s*, *t*) the mass of all the leaves between the point **r**(*s*, *t*) and the tip **r**(*ℓ*(*t*), *t*), and *ρ*(*σ*, *t*) the linear density at the point **r**(*σ*, *t*).

The term *linear* density indicates the mass per unit of length. This quantity is different from the mass per unit of volume, which we refer to as *volume* density and denote with *ρ*_3_. If we assume that the rod has a circular cross-section with radius *R*(*s*, *t*) at the point **r**(*s*, *t*), the relation between these two quantities is simply given by

$$\begin{array}{c} \rho \left(s,t\right)=\rho_{3}\left(s,t\right)\pi R^{2}\left(s,t\right). \end{array}$$

#### Modeling the shoot extension

We consider primary growth as the result of a “growth from the tip”, so we are assuming that the addition of mass occurs only at the tip. As a result of such a process, a point initially located at **r**(*S*, 0) for some *S* ∈ [0, *ℓ*_0_], reaches the point **r**(*s*, *t*). The arc-length parameter *s* ∈ [0, *ℓ*(*t*)] at time *t* is a function of the initial arc-length parametrization *S* ∈ [0, *ℓ*_0_]. Assuming that the local extension occurs with a factor *G*_0_, the function *s*(*S*, *t*) has to satisfy the following partial differential equation:

$$\begin{array}{c} \begin{array}{c} G\left(s,t\right)=\{\begin{array}{cc} G_{0} & \text{if}\;s\left(S,t\right)\in [\ell \left(t\right)-\ell_{g},\ell \left(t\right)]\\ 0 & \text{otherwise}, \end{array}\\ \left\{ \begin{array}{c} \partial_{t}\partial_{S}s\left(S,t\right)=G\left(s\left(S,t\right),t\right)\cdot \partial_{S}s\left(S,t\right)\\ s(S,0)=S\;,\;s(0,t)=0 \end{array}\right. \end{array} \end{array},$$ (2)

where *ℓ*_*g*_ represents the length of the zone at the tip at which the extension occurs.

#### Modeling the shoot rigidification

For modelling growing trees, the fundamental assumption is that the new layer of material formed during the secondary growth process do not affect the balance of the total forces and moments which were previously applied to the stem [33]. In other words, in an infinitesimal interval of time, the secondary growth does not affect the current configuration, which is described by the curvature ∂_*s*_*θ*, but affects the intrinsic configuration and its curvature *κ*. By applying the equations developed in [21] to our case (see Section B in S1 Text for further details, but also [20, 22, 33] for a biological and mechanical basis on the secondary growth), we get the following relation for the time evolution of the intrinsic curvature *κ*:

$$\begin{array}{c} \partial_{t}\kappa =-\frac{\partial_{t}B}{B}\left(\kappa -\partial_{s}\theta \right), \end{array}$$ (3)

#### Equation for the tropisms: Directed stimulus, proprioception and secondary growth

External and internal stimuli affect the time evolution of the intrinsic configuration. To model this behaviour taking the rigidification into account, we need to make an assumption on the shape of the cross-section. As anticipated in Section, in our model the cross-section is a circle of radius *R*(*s*, *t*) at the point **r**(*s*, *t*). We can then complete Eq (3) with the terms of perception of a generically directed stimulus and proprioception [16, 21]:

$$\begin{array}{c} \partial_{t}\kappa =\frac{Gv_{R}}{R^{2}}\left(\alpha \;\text{cos}\;\theta -\beta \;\text{sin}\;\theta \right)-G\gamma \partial_{s}\theta -\delta \frac{\partial_{t}B}{B}\left(\kappa -\partial_{s}\theta \right), \end{array}$$ (4)

where *α* and *β* represents the response to a directional stimulus, while *γ* represents the sensitivity with respect to the proprioception. We will refer to them as *sensing* parameters. *δ* is the parameter regulating the intensity of the radial expansion effect on the shoot development. *v*_*R*_ represents the radial expansion rate.

The notation *α* cos *θ* − *β* sin *θ* is equivalent to $\tilde{\beta }\;\text{sin}\left(\theta_{p}-\theta \right)$. Indeed, we can write the couple $\left(\alpha ,\beta \right)\in R^{2}$ in polar coordinates by defining

$$\begin{array}{c} \tilde{\beta }=\sqrt{\alpha^{2}+\beta^{2}}\;,\;\theta_{p}=\;\text{arctan}(\frac{\alpha }{\beta }). \end{array}$$

Hence, $\alpha =\tilde{\beta }\;\text{sin}\;\theta_{p}$, $\beta =\tilde{\beta }\;\text{cos}\;\theta_{p}$ and $\alpha \;\text{cos}\;\theta -\beta \;\text{sin}\;\theta =\tilde{\beta }\;\text{sin}\left(\theta_{p}-\theta \right)$.

#### Summary of the equations

We group the equations introduced in Sections—in the following system:

$$\begin{array}{c} \left\{ \begin{array}{c} \partial_{t}\partial_{S}s\left(S,t\right)=G\left(s\left(S,t\right),t\right)\cdot \partial_{S}s\left(S,t\right)\\ \partial_{s}\left[B\left(s,t\right)\left(\kappa \left(s,t\right)-\partial_{s}\theta \left(s,t\right)\right)\right]=[\int_{s}^{\ell (t)}\rho \left(\sigma ,t\right)d\sigma +m_{l}\left(s,t\right)]g\;\text{sin}\;\theta \left(s,t\right)\\ \begin{array}{cc} \partial_{t}\kappa \left(s,t\right)= & \frac{G\left(s,t\right)v_{R}\left(s,t\right)}{R{\left(s,t\right)}^{2}}\left(\alpha \;\text{cos}\;\theta \left(s,t\right)-\beta \;\text{sin}\;\theta \left(s,t\right)\right)-G\left(s,t\right)\gamma \partial_{s}\theta \left(s,t\right)\\ & -\delta \frac{\partial_{t}B\left(s,t\right)}{B(s,t)}\left(\kappa \left(s,t\right)-\partial_{s}\theta \left(s,t\right)\right) \end{array}\\ s(S,0)=S\;,\;s(0,t)=0\\ \theta \left(0,t\right)=\theta_{0}\\ \kappa \left(s,0\right)=0\;,\;\kappa \left(\ell \left(t\right),t\right)=\partial_{s}\theta \left(\ell \left(t\right),t\right) \end{array}.\right. \end{array}$$ (5)

Regarding the boundary conditions, we are assuming that the searcher shoot is clamped at the base at an angle *θ*_0_. As stated in Section, at the initial time the intrinsic configuration consists in a straight rod, which means *κ*(⋅, 0) ≡ 0. The condition *κ*(*ℓ*(*t*), *t*) = ∂_*s*_*θ*(*ℓ*(*t*), *t*) means that there is no extra weight at the tip, hence the curvature is the same in both the intrinsic and the current configuration. As discussed in Sections C and D in S1 Text, to solve numerically system (5), we first rewrite all the equations with respect to the Lagrangian coordinates (*S*, *t*). Then, the system is numerically solved by discretizing it in time via the backward Euler method and integrating it in space using the finite element method.

### Application to experimental data

#### Geometry and biomechanical properties of the samples

In a first experiment, we collected morphological and biomechanical data from five searcher shoots for each species. For each individual, we selected, as far as possible, the longest searcher shoots in a self-supporting state. In their natural position, we measured the reach (*cm*) and orientation (degrees from the horizontal) defined as a straight line from the base to the apex of the searcher shoot. The geometry of the shoot was described from the internodes of the main stem. For each successive internode, we measured its length and its median diameter obtained from the mean of two orthogonal measurements. Bending properties of the base of the searcher stem were measured using four-point bending tests on a stem segment constituted of several internodes [34]. Flexural rigidity *EI* (which is denoted with the function *B* in System (5)) and structural Young’s modulus *E*_*str*_ were calculated from applied bending forces plotted against maximum deflections. Up to five weights were applied manually and each deflection was measured by observation with a dissecting microscope on the apparatus. Weight increments were chosen according to the bending resistance of each sample. Weights were constituted of stainless-steel or brass ranging from 8 *g* (*C. guianense*) to 50 *g* (*T. jasminoides*). Span distances were defined as proportional to the mean elliptical diameter of the stem segment. The span support was 40 times greater than the diameter and was ranging from 107 *mm* to 218 *mm* for the longest. The load span was comprised between one-half and two-thirds of the span support and ranged from 67 *mm* to 120 *mm*. For three positions along the measured segment (basal, medial, apical), the vertical and horizontal diameter, *d*_*v*_ and *d*_*h*_, were measured and the means were then used to calculate the second moment of area *I* of the axis. The experimental measurements show that the difference between *d*_*v*_ and *d*_*h*_ is small, so it is reasonable to assume a circular cross-section. The structural Young’s modulus of the stem (*E*_*str*_) (*MN*/*m*^2^) was estimated by dividing measured values of *EI* by calculated values of *I* [35].

#### Extensional growth of the samples and radial expansion rate

In a second experiment, we monitored the shoot extension of young, self-supporting, searcher shoots over one week for 11 shoots of *T. jasminoides* and one month for 19 shoots of *C. guianense* (see section *Materials and Methods* in [29] for further information on the environmental conditions). Three dates at three days intervals were recorded for *T. jasminoides* and five dates at 7 days of interval for *C. guianense*. For both species, we applied the same protocol starting by defining a reference mark on the most basal node of each main stem. From this mark, we measured at each date: (i) the total length of the shoot (*cm*); (ii) the length of each successive internode up to and including the apex (*cm*). The natural organisation of the shoot into separate internodes allowed us to identify and measure which internodes elongated between two dates (see S2 Table).

The data on the radius from the first experiment (discussed in Section) and the measurements of the extension from the second experiment allow us to compute the radial expansion rate *v*_*R*_. To accomplish this measurement, we consider the geometrical structure of the samples from the first experiment, i.e. the internodes. For each sample, we enumerate the internodes starting from the base. At the base of the *i*-th internode, we assign its arc-length parameter, which we denote with *s*_*i*_. Hence, according to our notation, *s*_1_ is the arc length at the base of the first internode, so *s*_1_ = 0, and if *j* > *i*, then *s*_*j*_ > *s*_*i*_. Moreover, since *s*_*j*_ > *s*_*i*_, the point **r**(*s*_*i*_, *t*) is closer to the base than **r**(*s*_*j*_, *t*), hence more mature. Consider the situation in which *j* = *i* + 1. Let Δ*t*_*i*_ be the time it takes the plant to elongate by an amount equal to the length of the *i*-th internode, that is

$$\begin{array}{c} \Delta t_{i}=\frac{\text{length}\;\text{i-th}\;\text{internode}}{\text{extension}\;\text{rate}} \end{array}$$

Assuming that the maturation process depends merely on the distance from the tip (see Section below), this implies that the radius *R*(*s*_*i*+*i*_, *t*) is going to expand until reaching the value *R*(*s*_*i*+1_, *t* + Δ*t*_*i*_) = *R*(*s*_*i*_, *t*) after its distance from the tip has increased by the length of the *i*-th internode. Hence, to retrieve the expansion rate at the base of the *i*-th internode, we take the difference between the radii at its extremes and divide it by Δ*t*_*i*_:

$$\begin{array}{c} v_{R}\left(s_{i},t\right)=\frac{R\left(s_{i},t\right)-R\left(s_{i+1},t\right)}{\Delta t_{i}}. \end{array}$$

We retrieve the extension rate from the data of the second experiment, as explained in Section below.

#### Fitting of the model’s biomechanical functions

The data provided by the first experiment were to estimate volume density *ρ*_3_, radius *R*, radial expansion speed *v*_*R*_, flexural rigidity *B* and leaves mass *m*_*l*_. Such an estimate was specific for each shoot, that is, for each shoot, we retrieved the parameters of the above-mentioned functions. The data from the first experiment describe the biomechanical properties of the samples at a fixed time of their development. To get information about the time evolution of those properties, we assume that they depend only on the distance from the tip. Hence, we have *ρ*_3_(*s*, *t*) = *ρ*_3_(*ℓ*(*t*) − *s*), *R*(*s*, *t*) = *R*(*ℓ*(*t*) − *s*) and the same for *v*_*R*_, *B* and *m*_*l*_ (more precisely, the mass of a single leaf ${\hat{m}}_{l}$ as explained below). In particular, we assume that

$$\begin{array}{cc} \rho_{3}\left(x\right) & =a_{3}x^{2}+b_{3}x+c_{3}\\ R(x) & =a_{R}x^{2}+b_{R}x+c_{R}\\ v_{R}\left(x\right) & =a_{v}x^{2}+b_{v}x+c_{v}\\ B(x) & =\frac{a_{fr}}{1+\text{exp}\;(b_{fr}\cdot \left(c_{fr}-x\right))}\\ {\hat{m}}_{l}\left(x\right) & =\frac{a_{l}}{1+\text{exp}\;(b_{l}\cdot \left(c_{l}-x\right))} \end{array},$$

where *x* = *ℓ*(*t*) − *s* is the distance from the tip and ${\hat{m}}_{l}$ is the mass of a single leaf. The coefficients (*a*_3_, *b*_3_, *c*_3_), …, (*a*_*l*_, *b*_*l*_, *c*_*l*_) are retrieved by fitting the functions with the experimental data (see Figures A-B in S1 Text). We choose a sigmoid for the flexural rigidity *B* and the mass of a single leaf ${\hat{m}}_{l}$ because we assume that the greater the distance from the tip, the more their value increases. To retrieve the mass of the leaves *m*_*l*_ from the mass of a single leaf ${\hat{m}}_{l}$ at a certain point of the shoot **r**(*s*, *t*), it is sufficient to sum the single leaf masses at the internode basis whose arc-length is greater than *s*. To transpose this concept into an equation, we denote with *I*(*t*) the number of internodes in the shoot at time *t*, starting the enumeration from the base. For instance, if there are four internodes, *I*(*t*) = {1, 2, 3, 4}, where 1 represents the internode at the base and 4 is the internode at the tip. We denote with *s*_*i*_(*t*) the arc length of the basis *i*-th internode (as for the radial expansion rate case). Then, we write the equation for *m*_*l*_ as follows:

$$\begin{array}{c} m_{l}\left(s,t\right)=\sum_{\left\{i\in I\left(t\right)\;:\;s_{i}\left(t\right)\geq s\right\}}n_{i}^{l}\left(t\right)\cdot {\hat{m}}_{l}\left(s_{i}\left(t\right),t\right), \end{array}$$

where $n_{i}^{l}\left(t\right)$ is the number of leaves at the base of the *i*-th internode at time *t*. The numerical scheme used to simulate System (5) results to be stable with respect to this fitting procedure. Indeed, by changing the function for the flexural rigidity *B* from a sigmoid to a polynomial, the resulting simulation of the main stem does not display any significant change. See Section G in S1 Text for further details.

#### Estimate of the extension zone parameter *ℓ*_*g*_ and the local extension factor *G*_0_

We model the primary growth process as a growth from the tip, with an extension zone of length *ℓ*_*g*_ located at the apex and a local extension factor *G*_0_. We consider only the case in which the length of the shoot is greater than *ℓ*_*g*_ and consequently, the length of the shoot *ℓ*(*t*) follows a linear law (i.e. a constant extension rate $(\dot{\ell })\left(t\right)$, see for instance [32], Section 4.3.1). For this reason, we consider only the samples in which the extension process is involving just the apical part and not the whole shoot. Such a property can be verified by looking at the internodes extension: if the length of the internodes at the base is not changing, we can use the sample; otherwise, we discard it. We estimate the extension rate $\dot{\ell }\left(t\right)$ in *cm*/*day* by looking at the difference in the total length of the sample at different time intervals, dividing by the time intervals themselves, and taking the average. The analytical solution of System (2) (see Section C in S1 Text) leads to a relation between this estimate and the extension parameters, which consists of the following equation:

$$\begin{array}{c} \dot{\ell }\left(t\right)=G_{0}\ell_{g} \end{array}$$

Regarding *ℓ*_*g*_, for each shoot, we sum the lengths in *cm* of the extending internodes and average them. Since many random effects may have influenced the shoot extension over these periods (e.g. local light, temperature, herbivores, support finding, etc.), we decided to compute a single value of $\dot{\ell }\left(t\right)$ and *ℓ*_*g*_ for all the shoots of the same species. To do so, we took the average also over all the estimated $\dot{\ell }\left(t\right)$ and *ℓ*_*g*_. To retrieve the parameter *G*_0_, we divide the estimate for the extension rate $\dot{\ell }\left(t\right)$ by the estimate for the extension zone length *ℓ*_*g*_.

#### Estimate of the sensing parameters from reach and orientation

To estimate the sensing parameters (*α*, *β*, *γ*) for a fixed intensity *δ* of the radial expansion term (see Eq (4)), we employ the data about reach and orientation and the numerical solution of the model. More precisely, let **r**_*f*_(*α*, *β*, *γ*) the apical point **r**(*ℓ*(*T*), *T*) of the current configuration resulting from the numerical solution of system 5 at the final time *T* with sensing parameters (*α*, *β*, *γ*). Hence, the reach and orientation of the simulated shoot are

$$\begin{array}{cc} {\text{Reach}}_{s}\left(\alpha ,\beta ,\gamma \right) & =|r_{f}\left(\alpha ,\beta ,\gamma \right)|\\ {\text{Orient}}_{s}\left(\alpha ,\beta ,\gamma \right) & =\text{arctan}\;(\frac{r_{f,2}\left(\alpha ,\beta ,\gamma \right)}{r_{f,1}\left(\alpha ,\beta ,\gamma \right)}) \end{array},$$

where *r*_*f*,1_ and *r*_*f*,2_ are the components of **r**_*f*_ respectively along **e**_1_ and **e**_2_. Let Reach_*e*_ and Orient_*e*_ be the experimental reach and orientation for the sample considered by the simulation; we consider the parameter-dependent difference between simulated and experimental values with the function

$$\begin{array}{c} F\left(\alpha ,\beta ,\gamma \right)={\left({\text{Reach}}_{s}\left(\alpha ,\beta ,\gamma \right)-{\text{Reach}}_{e}\right)}^{2}+{\left({\text{Orient}}_{s}\left(\alpha ,\beta ,\gamma \right)-{\text{Orient}}_{e}\right)}^{2}. \end{array}$$

**Fig 2:**
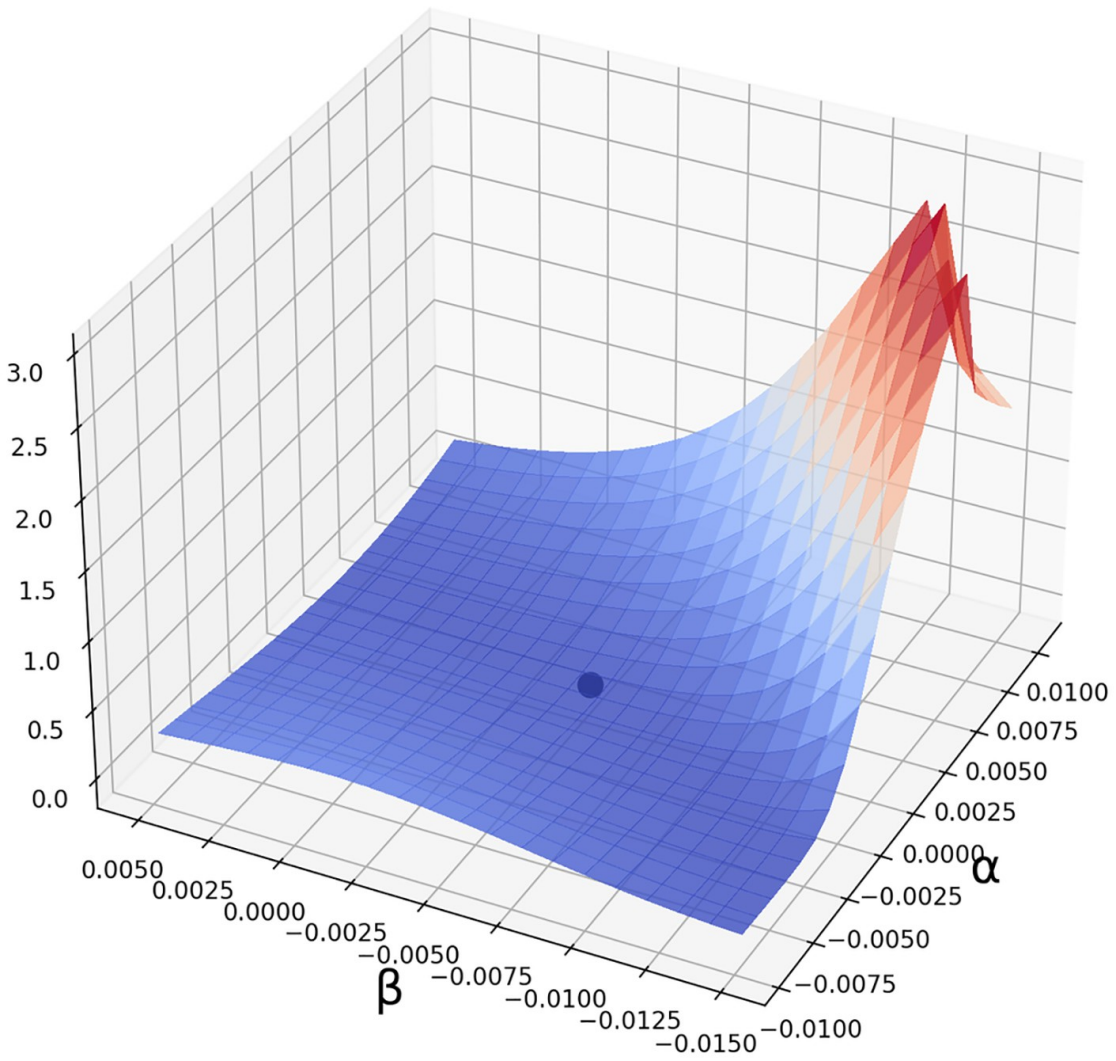
Graph of the function (*α*, *β*) → *F*(*α*, *β*, *γ*) for a sample of *T. jasminoides*. In this fgigure, *γ* is fixed at 0.008. The dot represents the minimum of the surface (approximately at *α* = 0.0005, *β* = −0.005). To estimate parameters (*α*, *β*, *γ*) we look for the minima of such surfaces for different values of gamma. Hence, we choose the minimum among those minima.

Hence, to estimate the sensing parameters, we fix an initial guess (*α*_0_, *β*_0_, *γ*_0_) and we minimize the value of *F* in a neighbourhood of that guess (see Fig 2).

## Results

**Fig 3:**
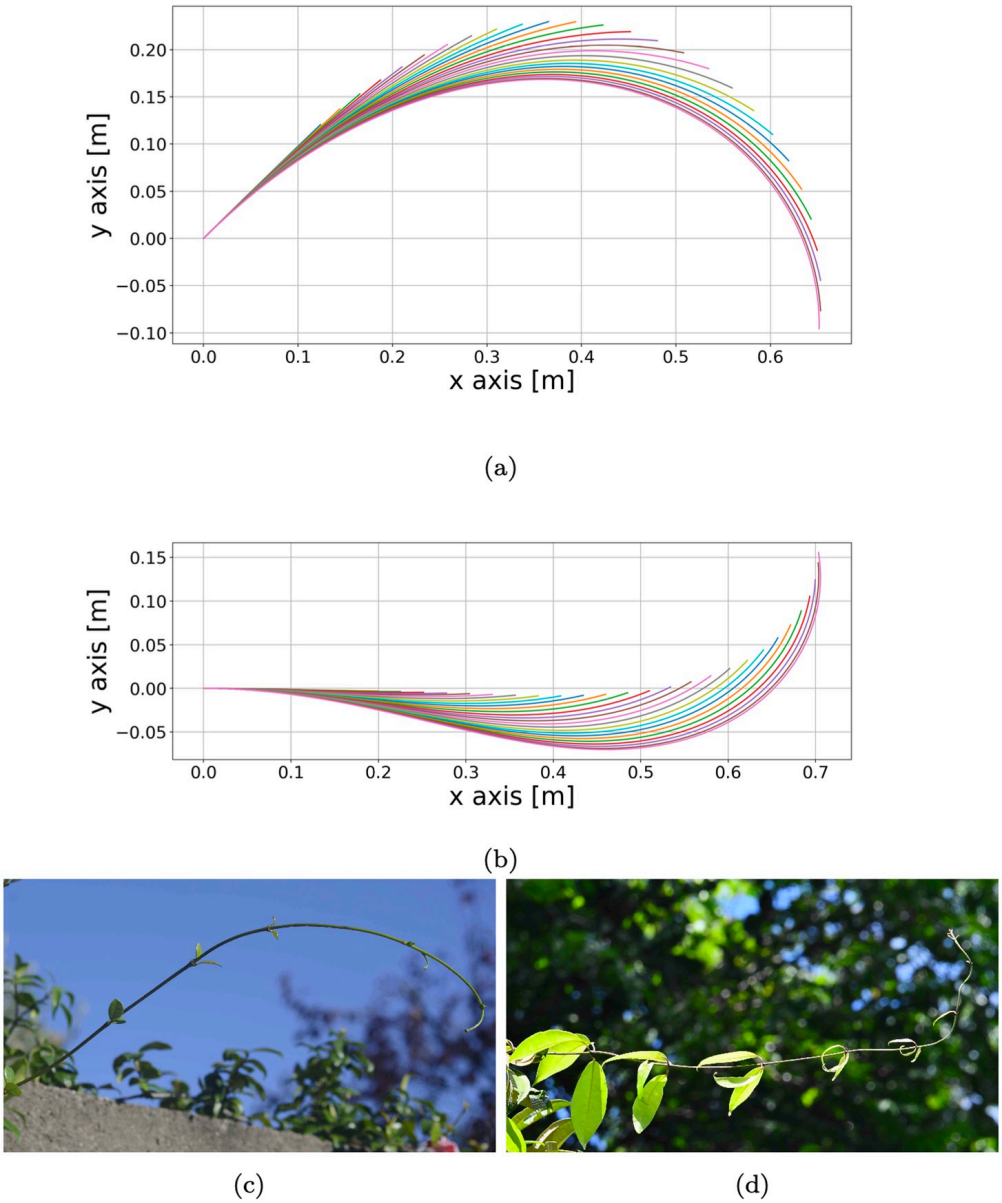
(3A),(3B): **Simulations for *T. jasminoides* and *C. guianense*, respectively**. The axes represent the (*x, y*)-coordinates of the simulation in the plane span{**e**_1_, **e**_2_}, where *x* is the coordinate along **e**_1_ and *y* the coordinate along **e**_2_. The different colours represent the different time steps of the simulation; each line of a single colour represents the main stem. Each stem has a fixed origin point at (0,0). The simulation of *T. jasminoides* has an initial inclination of *π*/4 with respect to the vertical line, while that of *C. guianense* has a horizontal initial inclination. In both cases, the plant develops horizontally. The behaviour of the *T. jasminoides* is due to its sensing activity, which displays a negative parameter *β*. In contrast, that of *C. guianense* droops because of its weight. (3C),(3D): **Images of the two samples of *T. jasminoides* and *C. guianense*, respectively, in their natural habitat**. These are the samples on which the numerical simulations are based. As one can observe, their behaviour resembles those displayed in Fig 3A and 3B: the *T. jasminoides* curls around towards itself, growing downwards; the *C. guianense* is strongly directed horizontally and points upwards with its tip.

We integrate system (5) numerically, calibrating the parameters for two specific climbing plant samples, one for *T. jasminoides* species, the other for *C. guianense* species (see Tables 1 and 2). For such simulations, the calibration of the sensing parameters (*α*, *β*, *γ*) is obtained by setting the radial extension parameter to *δ* = 0. The simulated stems are displayed in Fig 3. For *T. jasminoides*, the initial inclination of the stem with respect to the vertical line is *π*/4 radians. Initially, the stem grows upwards, but it is clear from the changes in the inclination of the tip that the growth direction is changing. Soon, the apical part of the shoot starts growing downwards. The case of *C. guianense* is different. The simulation displays a strong horizontal extension of the shoot. The initial inclination of the main stem is *π*/2 radians with respect to the vertical line, which means that, in the beginning, the shoot is directed horizontally. Looking at the time evolution displayed in Fig 3, we see that the tip is directed upward. Nevertheless, it is clear that in the simulation the whole *C. guianense* stem is drooping due to its weight.

**Table 1:** Estimated parameters for a sample of *T. jasminoides*.

Parameter	Value	Un. of Meas.	Index
*ℓ*_*g*_	1.4*e* − 01	*m*	*σ* : 3.3*e* − 02
$\dot{\ell }\left(t\right)$	2*e* − 02	*m* ⋅ *day*^−1^	*σ* : 5*e* − 03
*G*_0_	1.5*e* − 01	*day* ^−1^	−
(*a*_3_, *b*_3_, *c*_3_)	(1.2*e* + 04, −2.1*e* + 03, 1.6*e* + 03)	$\left(\frac{kg}{m^{5}},\frac{kg}{m^{4}},\frac{kg}{m^{3}}\right)$	*R*^2^ : 0.75
(*a*_*R*_, *b*_*R*_, *c*_*R*_)	(−6*e* − 04, 1.4*e* − 04, 6*e* − 04)	(*m*^−1^, −, *m*)	*R*^2^ : 0.97
(*a*_*v*_, *b*_*v*_, *c*_*v*_)	(1.3*e* − 04, 6*e* − 05, 2.6 − 05)	$\left(\frac{1}{day\cdot m},\frac{1}{day},\frac{m}{day}\right)$	*R*^2^ : 0.15
(*a*_*fr*_, *b*_*fr*_, *c*_*fr*_)	(1.6*e* − 02, 4.2, 6.8*e* − 1)	(*N* ⋅ *m*^2^, *m*^−1^, *m*)	*RMSD* : 7.5*e* − 04
(*a*_*l*_, *b*_*l*_, *c*_*l*_)	(5.8*e* − 05, 1*e* + 01, 1*e* − 01)	(*kg*, *m*^−1^, *m*)	*RMSD* : 3.4*e* − 05
(*α*, *β*, *γ*)	(5*e* − 04, −5*e* − 3, 8*e* − 3)	(*day*, *day*, −)	−
*θ*_0_	*π*/4	*radians*	−

The parameters (*a*_3_, *b*_3_, *c*_3_), …(*a*_*l*_, *b*_*l*_, *c*_*l*_) are specific for the sample, while the extension zone length *ℓ*_*g*_, the extension rate $\dot{\ell }\left(t\right)$ and the local extension factor *G*_0_ are averaged among different samples (see S2 Table for the growth data and S1 Text for the graphs of the fitting functions). In the “Index” column, we display the standard deviation from the average for *ℓ*_*g*_ and $\dot{\ell }(\text{t})$, while for the fitted parameters we display the *R*^2^ index or the root mean square deviation (*RMSD*) when we are using a non-linear regression.

**Table 2:** Estimated parameters for a sample of *C. guianense*.

Parameter	Value	Un. of Meas.	Index
*ℓ*_*g*_	1.7*e* − 01	*m*	*σ* : 3.5*e* − 02
$\dot{\ell }\left(t\right)$	2*e* − 02	*m* ⋅ *day*^−1^	*σ* : 2*e* − 03
*G*_0_	1.2*e* − 01	*day* ^−1^	-
(*a*_3_, *b*_3_, *c*_3_)	(6.1*e* + 01, −1.9*e* + 01, 1*e* + 03)	$\left(\frac{kg}{m^{5}},\frac{kg}{m^{4}},\frac{kg}{m^{3}}\right)$	*R*^2^ : 0.92
(*a*_*R*_, *b*_*R*_, *c*_*R*_)	(1.5*e* − 04, 9.6*e* − 04, 5.6*e* − 04)	(*m*^−1^, −, *m*)	*R*^2^ : 0.98
(*a*_*v*_, *b*_*v*_, *c*_*v*_)	(7.3*e* − 05, −6.9*e* − 05, 3.6*e* − 05)	$\left(\frac{1}{day\cdot m},\frac{1}{day},\frac{m}{day}\right)$	*R*^2^ : 0.11
(*a*_*fr*_, *b*_*fr*_, *c*_*fr*_)	(3.8*e* − 01, 4.1, 1.6)	(*N* ⋅ *m*^2^, *m*^−1^, *m*)	*RMSD* : 2*e* − 04
(*a*_*l*_, *b*_*l*_, *c*_*l*_)	(6.2*e* − 03, 5.7, 1.1)	(*kg*, *m*^−1^, *m*)	*RMSD* : 3.3*e* − 05
(*α*, *β*, *γ*)	(1*e* − 03, 4*e* − 03, 4*e* − 03)	(*day*, *day*, −)	−
*θ*_0_	*π*/2	*radians*	−

The parameters measured are equivalent to those of *T. jasminoides*, as explaned in the caption of Table 1.

To further investigate the role of the weight distributiuon along the stem, we numerically integrate a “weightless model” based just on the growing Eq (2) and on the following evolution equation of the curvature

$$\begin{array}{c} \partial_{t}\kappa =\frac{Gv_{R}}{R^{2}}\left(\alpha \;\text{cos}\;\theta -\beta \;\text{sin}\;\theta \right)-G\gamma \kappa . \end{array}$$ (6)

**Fig 4:**
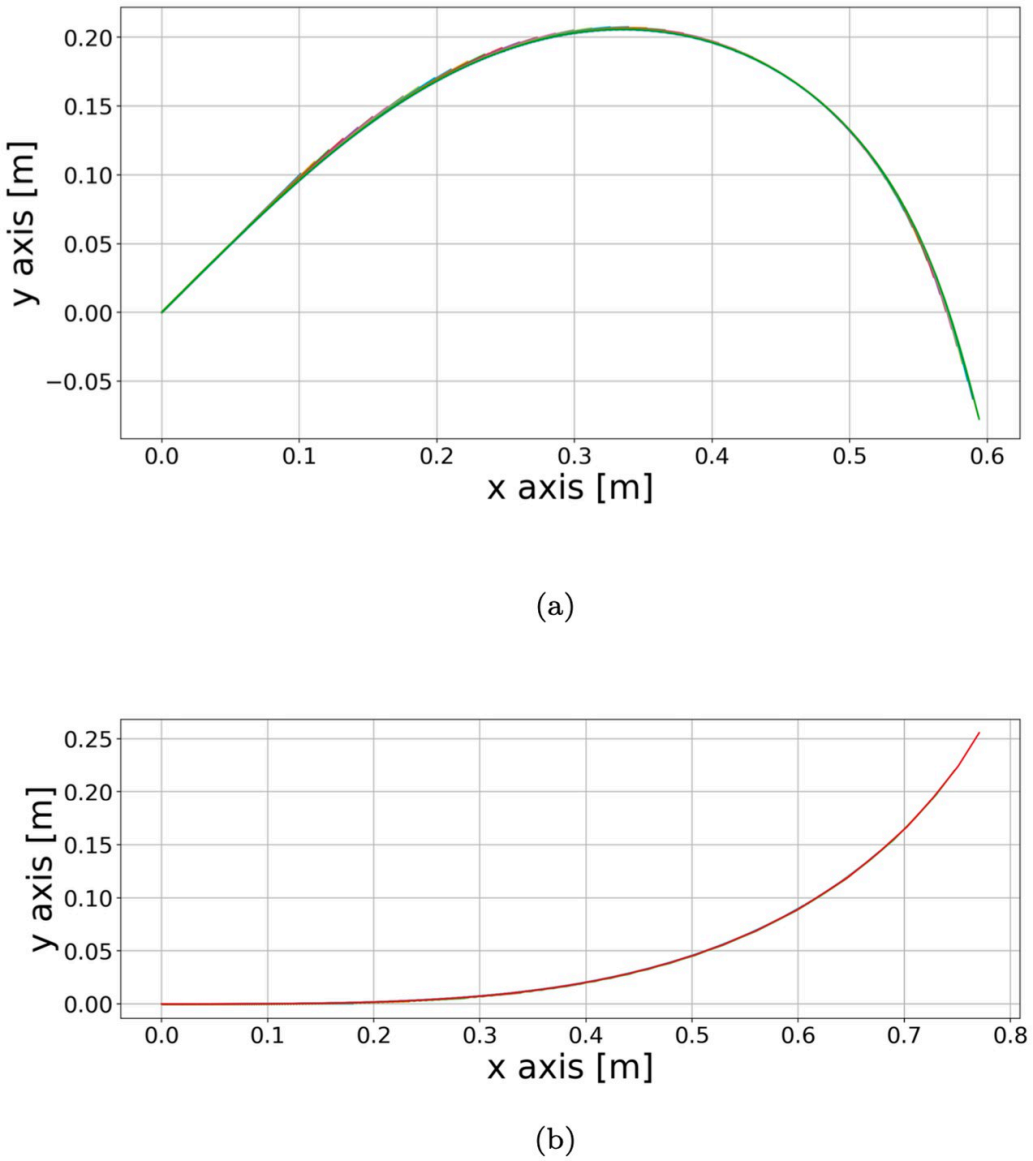
Simulations for *T. jasminoides* and *C. guianense* respectively. These simulations are based just on the growth Eqs (2) and (6), that is, they neglect the approximation of stem mechanics. The sensing parameters (*α*, *β*, *γ*) are optimised to obtain the best reach and orientation according to the experimental data. Such values are (6.8*e* − 03, −3*e* − 03, 2*e* − 02), (2.5*e* − 03, 2*e* − 03, 2*e* − 02), (in the ususal units of measure) respectively for the *T. jasminoides* and the *C. guianense*. We can observe that the simulation 4A for the *T. jasminoides* is growing downwards. The displayed behaviour is due to the sensing activity, in accordance with the result obtained for the simulation of the *T. jasminoides* in Fig 3A. On the other hand, the *C. guianense* is just growing upwards, differently from Fig 3B. This means that the mass can play a fundamental role in the formation of the plant shape.

We can extrapolate *G*_0_, *ℓ*_*g*_, *R* and *v*_*R*_ from the experimental data, calibrate the sensing parameters (*α*, *β*, *γ*) from the reach and the orientation of the shoot in consideration and set all these values as parameters in Eqs (2) and (6). The resulting simulations are displayed in Fig 4.

**Fig 5:**
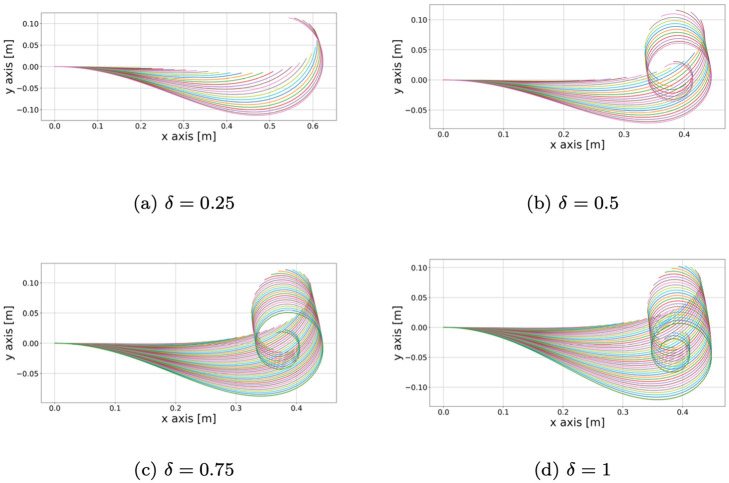
Simulations of *C. guianense* for increasing values of *δ*. Each of these simulations are obtained by following the procedure described in Section, so the sensing parameters are optimized to meet the experimental reach and orientation and may change among the simulations. We can observe the effect of the “negative proprioceptive” term (∂_*t*_*B*/*B*)∂_*s*_*θ* which amplifies the curling behaviour of the tip. This curling effect resembles the gravitropic “sign reversal” displayed in [24].

Considering again the model described by System (5), we fixed different values of *δ* and calibrated again the sensing parameters (*α*, *β*, *γ*) as described in Section. The parameters *G*_0_, *ℓ*_*g*_, *R* and *v*_*R*_ are based on the *C. guianense* dataset. The resulting simulations for different values of *δ* are displayed in Fig 5, in which we observe an increasing curling behaviour of the apex as *δ* increases.

## Discussion

### Interpretation and limitaitons of the result

The model developed in this study combines the primary growth of a searcher shoot (i.e. tip growth inducing stem extension) with the postural responses incorporating lateral expansion of the stem (i.e. secondary growth of the wood cylinder, thickening and maturation of primary tissues). These processes have been complemented by the variation of stem flexural rigidity *EI* (expressed by the function *B* in Eq (1)) and through the time development of the intrinsic curvature *κ*. As displayed in Section, the flexural rigidity was calibrated from the values of the (structural) Young’s modulus *E* observed at the base of the shoot, and from the series of diameters measured along the shoot to estimate the second moment of area *I*. In this procedure, *E* is considered to be constant in time and space. This simplification does not take into account the variations in *E* that exist due to the non-homogeneous tapering of tissues in the stem and their maturation. Although the variations along the climbing plant shoot of Young’s Modulus and second moment of area can well be of the same order, the variation of *R* plays a major role in the displayed simulations since it appears at the denominator of the sensing parameters in Eq (4).

In the simulations displayed in Fig 3, we observed that the stem is sagging. However, looking at the sensing parameters obtained in the fitting procedure (see Tables 1 and 2), we were able to distinguish whether the behaviour is due to the sensing activity of the shoot or to its weight. The negative *β* for the *T. jasminoides* expresses a downward stimulus which causes downward growth. So, we could conclude that the main driver of the *T. jasminoides* behaviour is the sensing activity. On the other hand, applying the same reasoning to the *C. guianense* stem, we reached the opposite conclusion. The positive *β* denotes a preference for upward growth, which explains the behaviour of the tip. However, as a consequence of its own weight, the main stem has a mostly horizontal development with a steep vertical change of growth in the part of the stem close to the tip.

Another evidence of the distinct role of the weight distribution along the stem in *T. jasminoides* and *C. guianense* behaviours are provided by the outcome of the simulations of the weightless model (displayed in Fig 4). A comparison between the two sets of simulations confirms the results on the role of the weight distribution along the shoots detailed previously. Indeed, as reported in Fig 4, the sensing parameter *β* for the weightless *T. jasminoides* is negative and the sensing activity orients the tip shoot downwards in a way that is similar (but not equal) to the case in which the weight affects the plant shoot development. On the other hand, the weightless model of *C. guianense* immediately grows upwards, without displaying a horizontal structure in the shoot portion before the apex.

### The role of the radial expansion parameter *δ*

In the study by Guillon et al. [21], it is assumed that the stem radial accretion does not change the local current curvature (see, e.g., the simulations in Fig 5 and related caption). Such an assumption led Guillon et al. [21] to add the term

$$\begin{array}{c} \frac{\partial_{t}B}{B}\left(\partial_{s}\theta -\kappa \right) \end{array}$$ (7)

in the sensing equation. The tendency to maintain the local current curvature rather than straighten the stem is in contrast with the stem proprioceptive activity. From a mathematical point of view, we can observe that the term $\frac{\partial_{t}B}{B}\partial_{s}\theta$ appearing in Eq (4) has an opposite sign with respect to the proprioceptive term, acting like a “negative proprioception” term in the sensing equation. Indeed, the ratio $\frac{\partial_{t}B}{B}$ is non-negative since the flexural rigidity *B* is both positive and increasing in time. Hence, if we consider the Eq (4) with *δ* > 0, it follows that the term $\delta \frac{\partial_{t}B}{B}\partial_{s}\theta$ has an opposite sign with respect to the proprioceptive term −*Gγ*∂*θ*. In particular, this means that the term $\frac{\partial_{t}B}{B}\partial_{s}\theta$ can mitigate (or destroy) the straightening effect of the proprioceptive term, introduced in [16] exactly to stabilize the sensing equation.

The occurrence of a “negative proprioception” is shown in the simulations in Fig 5, in which we can observe that the curling behaviour of the tip increases as the parameter *δ* increases. In particular, this behaviour affects the elongation direction of the tip, which grows downwards when it starts curling and then grows upwards again, resembling the gravitropic “sign reversal” displayed in [24].

The destabilising effect of the negative proprioception is confirmed by the spectral analysis of the operator involved in the simple equation developed by Bastien et al. [16]:

$$\begin{array}{c} \left\{ \begin{array}{c} \dot{\kappa }=-\beta \;\text{sin}\;\theta -\gamma \kappa \\ \theta^{\prime }=\kappa \end{array}\right. \end{array}$$ (8)

**Fig 6:**
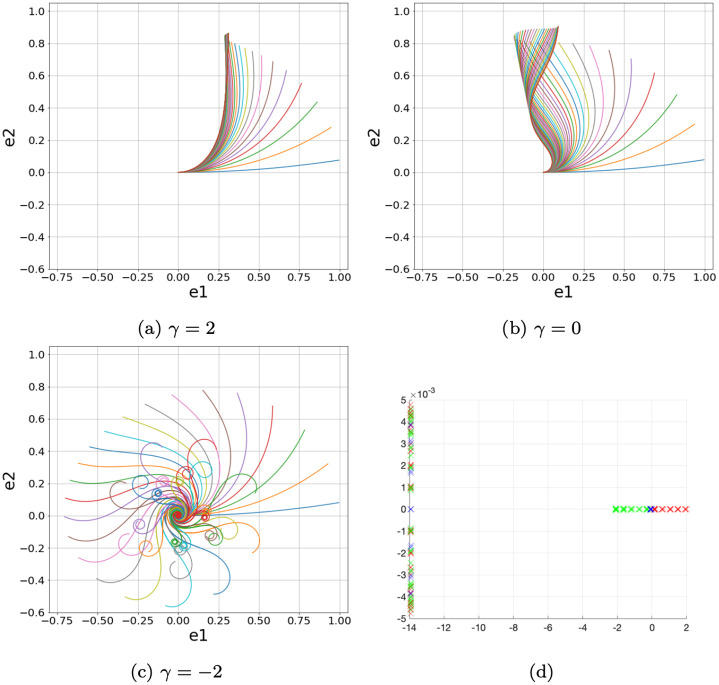
Simulations of the system (8) with *β* = 10 and (*S*, *T*) ∈ [0, 1] × [0, 2]. In Fig 6D, the eigenvalues for the linear operator in system (9). In green, the eigenvalues for *γ* > 0, in red for *γ* < 0 and in blue for *γ* = 0.

In this simple case, we observe that as the parameter varies, the stem dampens its oscillations around the vertical or not (see Fig 6). Linearizing the system around the equilibrium *θ* ≡ 0, we obtain the linear system

$$\begin{array}{c} \left[\begin{array}{cc} 0 & 0\\ 0 & 1 \end{array}][\begin{array}{c} \dot{\theta }\\ \dot{\kappa } \end{array}]=[\begin{array}{cc} \partial_{s} & -1\\ -\beta & -\gamma \end{array}][\begin{array}{c} \theta \\ \kappa \end{array}\right] \end{array}$$ (9)

With the help of a numerical simulation, we can compute the eigenvalues of the linear operator $T=[\begin{array}{cc} \partial_{s} & -1\\ -\beta & -\gamma \end{array}]$. The computed eigenvalues are displayed in Fig 6. We can observe that for *γ* ≥ 0 the real eigenvalues are negative, while when *γ* < 0 the real eigenvalues are positive. This means that in the negative proprioception case, there is an instability in the vertical equilibrium. This consideration is coherent with the behaviour of the steady state for system (9). Indeed, the steady state is given by

$$\begin{array}{c} \theta \left(s\right)=\theta_{0}e^{-\frac{\beta }{\gamma }s} \end{array}$$

Assuming *β* > 0, for *γ* > 0 the curve *θ*(*s*) tends to zero, which means that the correspondent stem tends to the vertical line. On the other hand, for *γ* < 0 *θ*(*s*) increases its values and consequently the corresponding stem tends to curl around itself.

Despite the destabilizing effect, it seems that a parameter *δ* ≃ 0.25 is more suited to describe the shape of the real *C. guianense* sample (see Fig 3). Indeed, as displayed in Fig 5, the tip tends to curl slightly in a similar way to Fig 3. This shows that our model provides a way to take secondary growth into account quantitatively with the parameter *δ*. However, given the dataset at our disposal, we cannot give an estimate of *δ*. Moreover, also due to its contrast with the proprioception, the estimate of *δ* represents a technical challenge and an interesting direction for future research.

### Biological interpretations

The application of the 2D model with *δ* ≈ 0 and calibrated with field data showed that a full description of primary and secondary growth processes as well as a description of the sensing activity was fundamental to reproducing the self-supporting growth of searcher shoots. Numerical simulations were able to reproduce similar maximal reach capacities as those observed in the field for both the samples in consideration. Simulations showed that both exhibited horizontal trajectories of self-supporting growth to attain their maximal reach capacities. 2D postures obtained at the end of the simulations were qualitatively equivalent to the ones observed in the field. Both species expressed a self-weight movement induced by mass distribution (in space) and accretion (in time). Nevertheless, they differed in terms of postural dynamics. These differences can be interpreted numerically by both sensing and mechanical parameters. From a sensing point of view, *C. guianense* exhibited a stronger anti-gravitropic behaviour than *T. jasminoides*. These explain the apical orientation of searcher shoots leading to an upward growth for *C. guianense* and a downward growth for *T. jasminoides*. From a mechanical point of view, *C. guianense* was more strongly effected than *T. jasminoides* in maintaining an upward growth in view of a faster mass accretion along the searcher shoot. However, these biological interpretations must be interpreted with caution with respect to the size of the dataset and observations that have been made under natural conditions without control over external factors. The samples considered in this work are not representative of all the variations we expect to see in nature. See for instance Section H in S1 Text for further photos of samples.

### Future improvements

The model developed here provides a theoretical and analytical framework that can be a useful tool to better understand the strategies of lianas to cross gaps with their searcher shoots under the effects of gravitational constraints. In particular, it allows us to see how the mass distribution, the stiffness and the length of the shoot influence its development and space orientation. To this purpose, the model parameters are fitted with the experimental data. As displayed in Section, for the *T. jasminoides* case this fitting procedure returns a negative parameter *β*, which is related to the vertical direction of the stimuli response activity. Since the climbing plant samples were not raised in a controlled environment, we cannot immediately interpret the negative value of *β* and the related behaviour of the simulation (Fig 3) with positive gravitropic behaviour. Nevertheless, the relation between the values of the sensing parameters and the observed behaviour of the climbing plant under unpredictable environmental conditions can be a matter of further studies.

Despite the uncontrolled environment and the basic measurements based on fully elongated shoots, the 2D model is revealed to be robust enough to reproduce the self-supporting state of the climbing plant species considered in this work. Moreover, the model can already take into account the tissue anatomical variations along the stems in terms of flexural rigidity, mass and radius, and how such variations affect the final climbing plant shape. Moving in the direction of a more in-depth study of the shoot structure, it may be interesting to investigate how the biomechanical state and biomass allocation change during the extension of a searcher shoot from a dynamic point of view. This would imply working on a large set of searcher shoots, regularly sacrificing some of them for destructive measurements, and reasoning in terms of chronosequence (i.e. by extrapolation), trying to work under conditions that are as homogeneous as possible in order to minimize the uncertainty factors (ontogenetic stage, environment, etc.). These measurements associated with the anatomical study of the tissues could allow a better understanding of the mechanism at the origin of the rigidification dynamics at all points of the searcher shoots. The anatomical organizations in the mechanical and postural life-histories of plants are potentially embracing ecology, biomechanics and robotics [36].

Searcher shoots are highly diverse in climbing plants and can have relatively complex structures depending on types of connected structures such as leaves, hooks, tendrils, branches, stem segments capable of circumnutatory movements, as well as intrinsic stem extension and postural dynamics [29]. The movements associated with these structures and generated by growth differentials within tissues are of significant importance for exploring and crossing spaces and attaching to supports. The model considered here in 2D is relatively simple and corresponds to an unbranched shoot with differential dynamics between internodes and leaves. The consideration of more complex shoot structures, involving variable mass load distributions in space and time and in a 3D volume is a higher step to properly simulate the self-supporting and searching mobility of searcher shoots in climbing plants. For instance, a 3D version of the model presented in this paper could be used to understand the role of circumnutation in climbing plant searching strategy. Another possible application of a 3D model could be to study how climbing plants change their mechanical internal properties when they encounter external obstacles to which they attach. All these problems cannot be addressed by using a 2D model and will be the subject of future studies.

### Conclusion

We formulated a model that takes secondary growth and proprioception into account. We showed how it is possible to estimate the parameters of the model from experimental data. Using the information about the extension of the stem, we computed the extension parameter *G*_0_ and the length of the extension zone *ℓ*_*g*_. Then, based on experimental data on the final reach and orientation of each stem, we calibrated the sensing parameters *α*, *β* and *γ*. Based on such measurements and on the related simulations in Fig 3, we were able to understand the role of the weight in the shapes observed in the photos in Fig 3.

For the two samples under consideration, we were able to say that the mechanical effect of the weight plays an important role in the shape of the *C. guianense*, while the downward growth of the *T. jasminoides* is mainly the consequence of its sensing activity. These conclusions are supported by the simulations based on a simplified model which considers just the plant sensing activity (see Fig 4). Such results confirm that the mechanical aspects in climbing plant searcher shoots, and, in particular, the variable linear density and the secondary growth are not negligible in order to obtain realistic climbing plant shapes in experimental data-based simulations.

## Supporting information

S1 Text Section A: Derivation of the mechanical model; Section B: Derivation of the secondary growth equation; Section C: Explicit solution to the extension equation; Section D: Numerical Integration of the model; Section E: Formulas used to retrieve the biomechanical properties of the samples; Section F: Fitting Figures; (G): Stability of the simulations. Figure A: Plot of the functions resulting from the fitting procedure for *T. jasminoides*. In blue we plot fitted curves for volume density, radius, radial expansion rate, flexural rigidity and leaves mass. Together with such plots, we display also the values of the experimental data and the curve of the absolute error. Figure B: Plot of the functions resulting from the fitting procedure for *C. guianense*. In blue we plot fitted curves for volume density, radius, radial expansion rate, flexural rigidity and leaves mass. Together with such plots, we display also the values of the experimental data and the curve of the absolute error. Figure C: (a)—(b): Simulations for *C. guianense* and *T. jasminoides* respectively, with polynomial flexural rigidity *B*. (c)—(d): Comparison between simulations displayed in Fig 3 of the main text with Figures Ca-Cb. The simulations obtained from the polynomial fitting are in magenta, while those with the sigmoid fitting are in green. Figure D: (a)—(b): two samples of *T. jasminoides*. In (a), the tip of the sample displays a negative gravitropic behaviour, while in (b) the sample has developed a main stem horizontally directed. (c)—(d): two samples of *C. guianense*. In (a), the main stem is only horizontally directed, while in (b), the tip is curling downwards.
(PDF)

S1 Table Experimental morphological data. (CSV)

S2 Table Experimental growth data. (ZIP)

S1 Code Code used for the simulations. Simulation of model (5) with null delta https://zenodo.org/record/8359924, simulation of model (5) with positive delta https://zenodo.org/record/8360202, simulation of the weightless model (6) https://zenodo.org/record/8360221.
(ZIP)
