# Supplementary material for: A 2D model to study how secondary growth affects the self-supporting behaviour of climbing plants
Source: PLoS Comput Biol. 2023 Oct 16;19(10):e1011538. doi: 10.1371/journal.pcbi.1011538 (PMC10602260; doi:10.1371/journal.pcbi.1011538)
Supplement: S1 Text — Section A: Derivation of the mechanical model; Section B: Derivation of the secondary growth equation; Section C: Explicit solution to the extension equation; Section D: Numerical Integration of the model; Section E: Formulas used to retrieve the biomechanical properties of the samples; Section F: Fitting Figures; (G): Stability of the simulations. Figure A: Plot of the functions resulting from the fitting procedure for T. jasminoides. In blue we plot fitted curves for volume density, radius, radial expansion rate, flexural rigidity and leaves mass. Together with such plots, we display also the values of the experimental data and the curve of the absolute error. Figure B: Plot of the functions resulting from the fitting procedure for C. guianense. In blue we plot fitted curves for volume density, radius, radial expansion rate, flexural rigidity and leaves mass. Together with such plots, we display also the values of the experimental data and the curve of the absolute error. Figure C: (a)—(b): Simulations for C. guianense and T. jasminoides respectively, with polynomial flexural rigidity B. (c)—(d): Comparison between simulations displayed in Fig 3 of the main text with Figures Ca-Cb. The simulations obtained from the polynomial fitting are in magenta, while those with the sigmoid fitting are in green. Figure D: (a)—(b): two samples of T. jasminoides. In (a), the tip of the sample displays a negative gravitropic behaviour, while in (b) the sample has developed a main stem horizontally directed. (c)—(d): two samples of C. guianense. In (a), the main stem is only horizontally directed, while in (b), the tip is curling downwards. (PDF) [file pcbi.1011538.s001.pdf]

Supporting Information for "A 2D Model to Study how the secondary growth affects the self-supporting behaviour of climbing plants": details on the model, fitting figures

Giacomo Vecchiato[1], Tom Hattermann[2], Michele Palladino[1,3], Fabio Tedone[1], Patrick Heuret[2], Nicholas P. Rowe[2], and Pierangelo Marcati[1]

[1]Gran Sasso Science Institute, L'Aquila, Italy
[2]AMAP, Univ Montpellier, CIRAD, CNRS, INRAe, IRD, Montpellier, France
[3]DISIM, Department of Information Engineering, Computer Science and Mathematics, University of L'Aquila, Via Vetoio - 67100 L'Aquila, Italy

Contents

A Derivation of the mechanical model **2**

B Derivation of the secondary growth equation **2**

C Explicit solution to the extension equation **3**

D Numerical Integration of the Model **4**

E Formulas used to retrieve the biomechanical properties of the samples **5**

F Fitting Figures **5**

G Stability of the simulations **8**

H Variability of behaviours **10**

A Derivation of the mechanical model

Let $\mathbf{r}(s,t)$ denote the position in $\mathbb{R}^2$ of the point of the shoot that at time t has arch length s for the current configuration, as defined in the main text. $\mathbf{e}_1$ and $\mathbf{e}_2$ we indicate respectively the horizontal and the vertical orthonormal axes, hence the gravity acceleration is $-g \cdot \mathbf{e}_2$. We recall that we are assuming that the time scale for the mechanical equilibrium is much shorter than the time for the biological growth of the plant. Hence, the Kirchhoff equations for the balance of forces and moments read the following form:

$$\begin{aligned} &\mathbf{n}(s,t) + \int_s^{\ell(t)} \mathbf{f}(\sigma,t) d\sigma = 0 \\ &\partial_s m(s,t) + \partial_s r_1(s,t) n_2(s,t) - \partial_s r_2(s,t) n_1(s,t) = 0 \end{aligned} \tag{1}$$

(we are considering $\mathbf{n}(\ell(t),t) = 0$, which means no internal forces developed at the terminal point of the shoot). Here m represents the internal moment of force, $\mathbf{n}$ the internal force and $\mathbf{f}$ the external force per unit length. The subscripts 1 and 2 represent the components of a vector with respect to $\mathbf{e}_1$ and $\mathbf{e}_2$ respectively. The term

$$\int_s^{\ell(t)} \mathbf{f}(\sigma,t) d\sigma$$

represents the force acting on the stem portion from $\mathbf{r}(s,t)$ to $\mathbf{r}(\ell(t),t)$. Since we are considering just gravity without external loads, we have the mass of the main stem, which gives the force term $-\rho(s,t)g$ and the mass of the leaves above $\mathbf{r}(s,t)$, which gives $m_l(s,t)g$. The direction of the gravity force is along $\mathbf{e}_2$. Hence, substituting these quantities in the equations (1) we obtain:

$$\begin{aligned} \mathbf{n}(s,t) &= -g \left[\int_s^{\ell(t)} \rho(\sigma,t) d\sigma + m_l(s,t) \right] \mathbf{e}_2 \\ \frac{\partial \mathbf{m}}{\partial s}(s,t) &= \sin\theta(s,t)\, g \left[\int_s^{\ell(t)} \rho(\sigma,t) d\sigma + m_l(s,t) \right] \mathbf{e}_3 \end{aligned} .$$

Now, we consider the constitutive law $m(s,t) = -B(s,t)[\partial_s \theta(s,t) - \kappa(s,t)]$, which leads to the equation

$$\left[\int_s^{\ell(t)} \rho(\sigma,t) d\sigma + m_l(s,t) \right] g \sin\theta(s,t) = \partial_s [B(s,t)(\kappa(s,t) - \partial_s \theta(s,t))]. \tag{2}$$

B Derivation of the secondary growth equation

In this section, we retrieve equation (3) of the main text. We recall that we are assuming that the new layer of material formed during the secondary growth process does not affect the balance of the total forces and moments which were previously applied to the stem. Using a formal mathematical description, this

means that the infinitesimal mass accretion due to the secondary growth does not affect the current curvature $\partial_s\theta$, but it changes only the intrinsic curvature κ. The two curvatures are related by the usual constitutive relation, which we rewrite as follows

$$\partial_s\theta = \kappa - \frac{m}{B}$$

the secondary growth affects κ, B, but not $\partial_s\theta$ nor m (because it is part of the balance of moments acting on the plant). So, considering just the secondary growth process and differentiating with respect to time, we get

$$0 = \partial_t\kappa + \frac{m}{B^2}\partial_t B$$

substituting again the constitutive relation for m, we get the equation

$$\partial_t\kappa = -\frac{\partial_t B}{B}(\kappa - \partial_s\theta)$$

C Explicit solution to the extension equation

We solve the equations for the extension, represented by the following system

$$\begin{array}{l} G(s,t) = \begin{cases} G_0 & \text{if } s(S,t) \in [\ell(t) - \ell_g, \ell(t)] \\ 0 & \text{otherwise,} \end{cases} \\ \begin{cases} \partial_t\partial_S s(S,t) = G(s(S,t),t)\cdot\partial_S s(S,t) \\ s(S,0) = S\,,\; s(0,t) = 0 \end{cases} \end{array} . \tag{3}$$

We recall that we are considering the case $\ell_g \leq \ell_0$. We can rewrite 3 as follows

$$\partial_t[\ln\partial_S s] = G,$$

which leads to

$$s(S,t) = \int_0^S e^{\int_0^t G(\sigma,\tau)d\tau}d\sigma,$$

where, with a slight abuse of notation, we are identifying $G(s(S,t),t)$ with $G(S,t)$. Since G is non-negative, we can deduce that s is an increasing function both in space and time, in particular, it is strictly increasing in space. Define $P(t)$ that value in $[0,\ell_0]$ such that $s(P(t),t) = \ell(t) - \ell_g$. Since s is strictly increasing in space, $P(t)$ is uniquely well-defined. Integrating in time (3), we obtain:

$$\partial_S s(S,t) = \begin{cases} \varphi_1(S) & S \in [0,P(t)) \\ e^{tG_0}\varphi_2(S) & S \in (P(t),\ell_0] \end{cases} \tag{4}$$

For some functions $\varphi_1 : [0,\ell_0] \to [0,+\infty)$, $\varphi_2 : [\ell_0 - \ell_g, \ell_0] \to [0,+\infty)$. Since for $t = 0$ we have $\partial_S s(S,0) = 1$, we deduce that $\varphi_2 = 1$, and $\varphi_1 = 1$ in $[0,\ell_0 - \ell_g]$.

Calling $\phi(S) = \int_{\ell_0-\ell_g}^{S} \varphi_1(\sigma) d\sigma$ and integrating in space equation (4) we have:

$$s(S,t) = \begin{cases} S & S \in [0, \ell_0 - \ell_g] \\ \ell_0 - \ell_g + \phi(S) & S \in [\ell_0 - \ell_g, P(t)) \\ \ell_0 - \ell_g + \phi(P(t)) + (S - P(t))e^{tG_0} & S \in (P(t), \ell_0] \end{cases} \tag{5}$$

By definition $s(P(t), t) = \ell(t) - \ell_g$, hence we find the equality $\phi(P(t)) = \ell(t) - \ell_0$. Substituting this value in the third relation expressed in (5) and using the fact that $\ell(t) = s(\ell_0, t)$, we obtain:

$$P(t) = \ell_0 - \frac{\ell_g}{e^{tG_0}}$$

$P(t)$ is a generic point $S \in [\ell_0 - \ell_g, \ell_0)$, in particular $P(t) = S$ when $e^{tG_0} = \frac{\ell_g}{\ell_0 - S}$. So when we impose continuity for $\partial_S s(S,t)$, we find the expression for φ_1:

$$\varphi_1(S) = \frac{\ell_g}{\ell_0 - S} \text{ for } S \in [\ell_0 - \ell_g, \ell_0]$$

Hence, s is:

$$s(S,t) = \begin{cases} S & S \in [0, \ell_0 - \ell_g] \\ \ell_0 - \ell_g + \ell_g \ln \frac{\ell_g}{\ell_0 - S} & S \in [\ell_0 - \ell_g, \ell_0 - \frac{\ell_g}{e^{tG_0}}] \\ \ell_0(1 - e^{tG_0}) + \ell_g G_0 t + S e^{tG_0} & S \in [\ell_0 - \frac{\ell_g}{e^{tG_0}}, \ell_0] \end{cases} \tag{6}$$

This function is $C^1([0, \ell_0] \times [0, +\infty); [0, +\infty))$ and it is increasing in space and time.

D Numerical Integration of the Model

The explicit expression of $s(S,t)$ discussed in Section C lets us write a function w defined on $[0, \ell(t)] \times [0, T]$ as a function of $(S,t) \in [0, \ell_0] \times [0, T]$. We define $\tilde{w}(S,t) = w(s(S,t), t)$. The relations between the partial derivatives is given by the following equations:

$$\partial_s w(s(S,t), t) = \frac{1}{\partial_S s(S,t)} \partial_S \tilde{w}(S,t)$$
$$\partial_t w(s(S,t), t) = \partial_t \tilde{w}(S,t) - \partial_S \tilde{w}(S,t) \frac{\partial_t s(S,t)}{\partial_S s(S,t)}.$$

Omitting the dependence from (S,t) and dropping the "$\sim$", we recast System (5) of the main text as follows:

$$\begin{cases} \partial_t \kappa - \partial_S \kappa \frac{\partial_t s}{\partial_S s} = \frac{G v_R}{R^2}(\alpha \cos\theta - \beta \sin\theta) - G\gamma \frac{1}{\partial_S s} \partial_S \theta - \delta \frac{B_t}{B} \left(\kappa - \frac{1}{\partial_S s} \partial_S \theta \right) \\ \partial_S s \, (\mu + m_l) g \sin\theta = \partial_S \left[B(\kappa - \frac{1}{\partial_S s} \partial_S \theta) \right] \\ \partial_S \mu = -\rho \partial_S s \end{cases} \tag{7}$$

Where B_t is the known function $\partial_t B$ of equation (5) of the main text. System (7) is then integrated by discretizing the time via backward Euler method and integrating in S using the finite elements method.

E Formulas used to retrieve the biomechanical properties of the samples

We use the notations introduced in Section 2.2.1 of the main text. In the four-point bending test, the flexural rigidity B is calulated with the following formula

$$a = \frac{L-l}{2}$$
$$B = b\frac{L^3}{48}\frac{3a}{L} - 4\left(\frac{a}{L}\right)^3,$$

where l is the load support (i.e. the distance between the two internal supports), L is the span of the support (i.e. the distance between the two external panniers) and b is the slope of the fore-deflection curve. Regarding the second moment of area I of the axis, it is measured with the formula

$$I = \frac{\pi}{4}\left(\frac{d_v}{2}\right)^3\frac{d_h}{2}.$$

F Fitting Figures

In this section we display the figures resulting from the fitting procedure for the functions displayed in Section 2.2.3 of the main text.

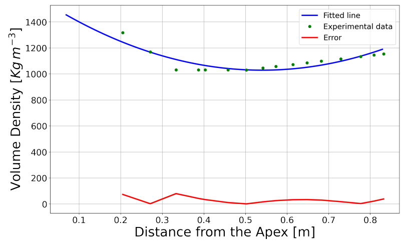

(a)

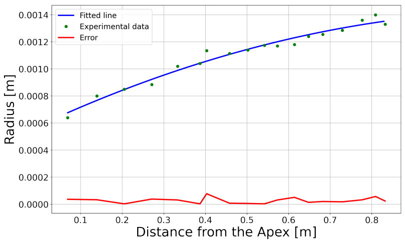

(b)

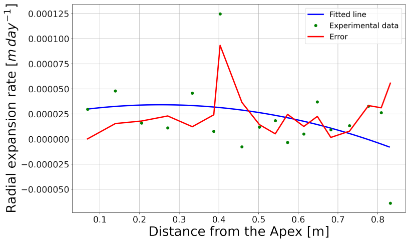

(c)

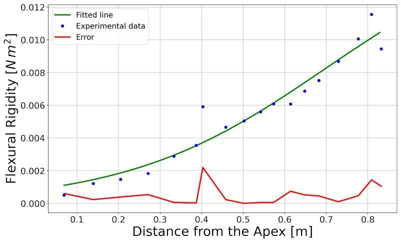

(d)

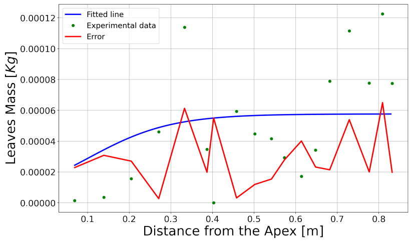

(e)

Figure A: Plot of the functions resulting from the fitting procedure for *T. jasminoides*. In blue we plot fitted curves for volume density, radius, radial expansion rate, flexural rigidity and leaves mass. Together with such plots, we display also the values of the experimental data and the curve of the absolute error.

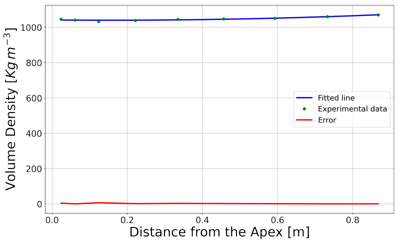

(a)

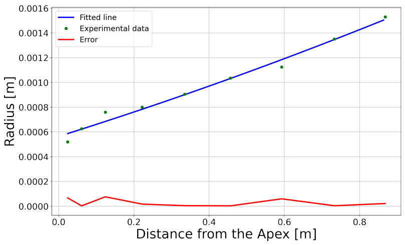

(b)

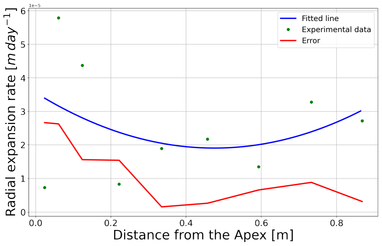

(c)

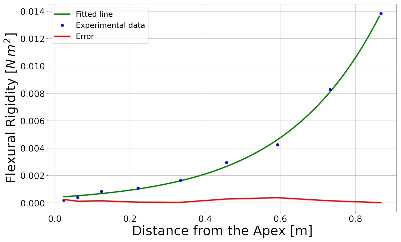

(d)

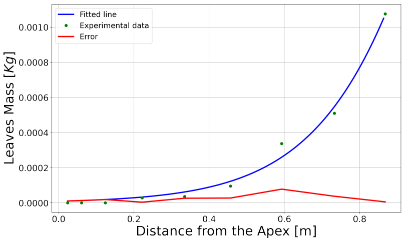

(e)

Figure B: Plot of the functions resulting from the fitting procedure for *C. guianense*. In blue we plot fitted curves for volume density, radius, radial expansion rate, flexural rigidity and leaves mass. Together with such plots, we display also the values of the experimental data and the curve of the absolute error.

G Stability of the simulations

In Figure C, we display the numerical simulations of System (5) of the main text in which the flexural rigidity is fitted with a second-order degree polynomial instead of a sigmoid:

$$B = a_{fr}x^2 + b_{fr}x + c_{fr}$$

The simulations of the main stem development for polynomial cases are close to the sigmoid one. This means that the numerical scheme used to solve the system is stable with respect to the fitting procedure.

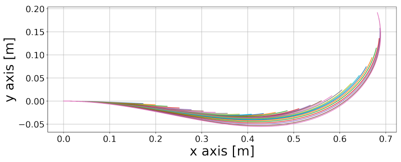

(a)

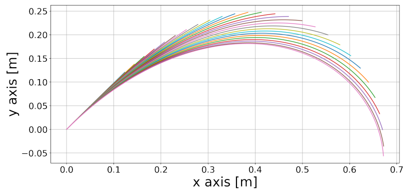

(b)

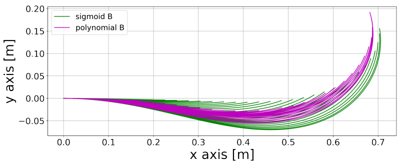

(c)

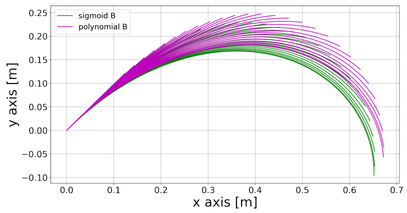

(d)

Figure C: (Ca) - (Cb): Simulations for *C. guianense* and *T. jasminoides* respectively, with polynomial flexural rigidity B.
(Cc) - (Cd): Comparison between simulations displayed in Fig 3 of the main text with Figs Ca - Cb. The simulations obtained from the polynomial fitting are in magenta, while those with the sigmoid fitting are in green.

H Variability of behaviours

In Fig D we display how the behaviour of *T. jasminoides* and *C. guianense* in their natural habitat can be different from that displayed in Fig 3 c-d of the main text.

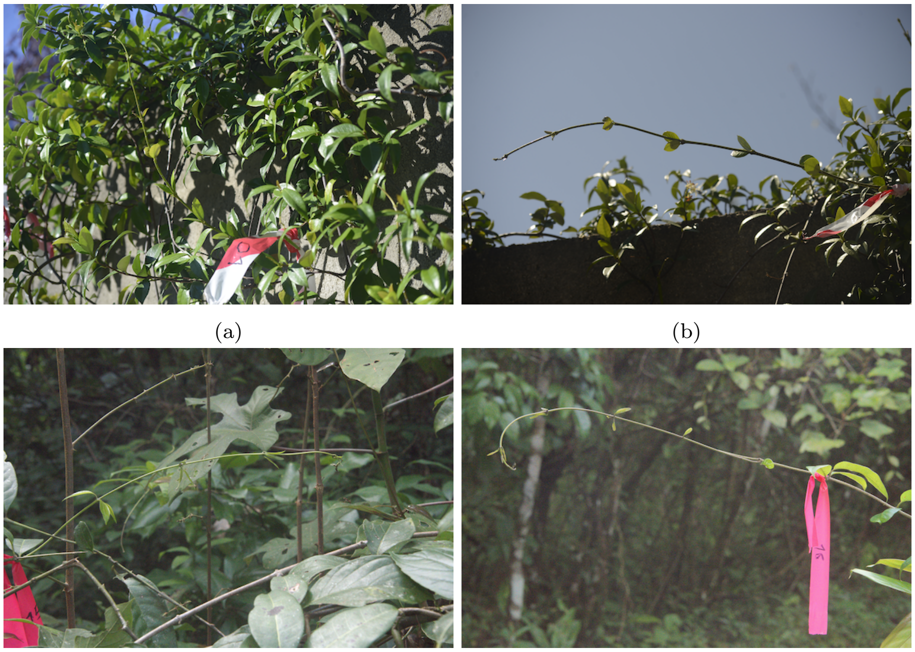

(a) (b) (c) (d)

Figure D: (Da)-(Da): two samples of *T. jasminoides*. In Da, the tip of the sample displays a negative gravitropic behaviour, while in Db the sample has developed a main stem horizontally directed.
(Dc)-(Dd): two samples of *C. guianense*. In Dc, the main stem is only horizontally directed, while in Dd, the tip is curling downwards.
